# Dose–Response Relationship between High-Fidelity Simulation and Intensive Care Nursing Students’ Learning Outcomes: An Italian Multimethod Study

**DOI:** 10.3390/ijerph19020617

**Published:** 2022-01-06

**Authors:** Angelo Dante, Carmen La Cerra, Valeria Caponnetto, Vittorio Masotta, Alessia Marcotullio, Luca Bertocchi, Fabio Ferraiuolo, Cristina Petrucci, Loreto Lancia

**Affiliations:** 1 Department of Health, Life and Environmental Sciences, University of L’Aquila, Rita Levi Montalcini Building, Via G. Petrini, 67100 L’Aquila, Italy; angelo.dante@univaq.it (A.D.); carmen.lacerra@guest.univaq.it (C.L.C.); vittorio.masotta1@graduate.univaq.it (V.M.); alessia.marcotullio@graduate.univaq.it (A.M.); luca.bertocchi@graduate.univaq.it (L.B.); fabio.ferraiuolo@graduate.univaq.it (F.F.); cristina.petrucci@univaq.it (C.P.); loreto.lancia@univaq.it (L.L.); 2 Department of Applied Clinical Sciences and Biotechnology, University of L’Aquila, Via Vetoio, 67100 L’Aquila, Italy

**Keywords:** high-fidelity simulation, graduate nursing students, critical care nurses, critical care nurse competences, learning outcomes

## Abstract

*Background*: The best application modality of high-fidelity simulation in graduate critical care nursing courses is still rarely investigated in nursing research. This is an important issue since advanced nursing skills are necessary to effectively respond to critically ill patients’ care needs. The aim of the study was to examine the influence of a modified teaching model based on multiple exposures to high-fidelity simulations on both the learning outcomes and the perceptions of graduate students enrolled in a critical care nursing course. *Methods*: A multimethod study involving a sample of graduate critical care nursing students was conducted. A theoretical teaching model focused on multiple exposures to high-fidelity simulations is currently applied as a teaching method in an Italian critical care nursing course. According to the Kirkpatrick model for evaluating training programs, the performance, self-efficacy, and self-confidence in managing critically ill patients were considered learning outcomes, while satisfaction with learning and students’ lived experiences during the experimental phases were considered students’ perceptions. *Results*: Multiple exposures to high-fidelity simulations significantly improved performance, self-efficacy, and self-confidence in managing virtual critically ill patients’ care needs. The satisfaction level was high, while lived experiences of participants were positive and allowed for better explanation of quantitative results of this study. *Conclusions*: Multiple exposures to high-fidelity simulations can be considered a valuable teaching method that can improve the learning outcomes of graduate nurses enrolled in an intensive care course.

## 1. Introduction

For some years now, there has been an ongoing interest in documenting the impact of high-fidelity simulations on nursing students’ learning outcomes [1,2]. This technology-based educational method, performed through an interactive patient simulator able to reproduce life-like clinical conditions, offers students the opportunity to apply their theoretical knowledge in a safe environment that faithfully reproduces clinical reality [3]. Integrating high-fidelity simulations in traditional educational programs allows faculties to fulfill the educational needs of students, making it possible for them to improve their technical skills and academic performance, assimilate appropriate behaviors, manage their emotions, strengthen their critical thinking, and effectively face unexpected events in a safe setting [4,5,6]. In this regard, a growing number of studies have confirmed the positive impact of high-fidelity simulations on undergraduate nursing students’ learning outcomes [7,8], while, for graduate students, the issue is still open, and further evidence is needed [1,9].

To guarantee the highest learning outcomes regarding graduate critical care nursing courses, faculty members need to be informed about the ideal number of simulation sessions, the optimal length of each session, the best facilitation methods, and the ideal number of days before a subsequent session to ensure learning retention [1,2,10]. In addition, the faculty needs to know if the conventional simulation modality based on briefing, simulation, and debriefing can serve as an empirical reference in postgraduate critical care nursing education [11].

In this framework, achieving satisfactory learning gains could be challenging for faculty, especially considering that graduate students generally have better clinical competence than their undergraduate colleagues and a greater need for further clinical competencies, making them able to face more complex clinical conditions [1,9]. For these reasons, to improve the clinical competence of graduate nurses enrolled in a critical care course, very realistic scenarios, a high level of student engagement, educators with deep clinical experience, and serious organizational efforts are strongly required [12,13,14,15,16].

It is well known that critical care nurses are called upon to operate in dynamic healthcare fields, as they care for patients at high risk of clinical impairment and, therefore, should be able to provide invasive interventions in a safely and timely way, as well as respond to the patient family’s needs [17,18]. High-fidelity simulations represent a valuable tool that could contribute to the development of an advanced level of clinical competence in graduate educational programs [19,20]. For this reason, it is crucial to fuel debate about the most suitable application modality for this tool, with the final aim of improving the learning outcomes of graduate nurses enrolled in a critical care course.

For the study hypothesis, it was assumed that multiple exposures to high-fidelity simulations beneficially impact nurses’ learning outcomes and their lived experiences.

This study aimed to investigate the impact of a modified teaching model based on multiple exposures to high-fidelity simulations on the learning outcomes and perceptions of graduate students enrolled in an intensive care nursing program.

## 2. Materials and Methods

### 2.1. Study Design, Setting, and Participants

A multimethod sequential study (QUAN → QUAL), in which the quantitative and qualitative approaches were considered equivalent [21], was conducted at the University of L’Aquila, Italy. After a Bachelor of Science in Nursing, Italian graduate nurses can decide to continue their studies by applying for a Master of Science in Nursing (lasting two years) or specialization courses (lasting 1 year). This study was conducted during the 2020–2021 academic year involving 21 graduate nurses enrolled in a specialization course, i.e., an intensive care program. Aspirants who want to attend the intensive care program must be graduate nurses. No additional prerequisites are required; however, if the number of candidates exceeds the number of available places, aspirants must complete an admission test.

The intensive care program aims at providing students with advanced technical and nontechnical skills to manage life-threatening conditions. It lasts 1500 h/year (60 credits; 1 credit = 25 h), which is subdivided into 750 h of lessons, 550 h of clinical training, and 200 h of other learning activities. During their clinical training, students must participate in educational activities based on high-fidelity simulations to learn how to manage dangerous and infrequent life-threatening clinical conditions.

Since, in this study, all students enrolled in the intensive care course were necessarily graduate nurses, the terms ‘nurses’, ‘graduate nurses’, and ‘students’ were used interchangeably.

### 2.2. Learning Model and Experiences

**Figure 1:**
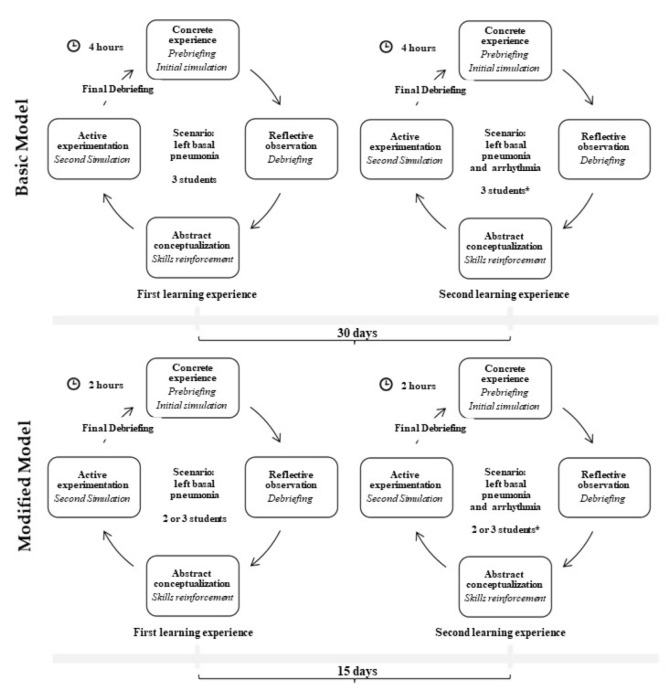
Basic and modified teaching models for simulation activities (* group composition randomly changed).

At the experimental simulation laboratory of the University of L’Aquila, a teaching model based on multiple exposures to high-fidelity simulations was previously developed and utilized to improve graduate nurses’ skills in the management of acute respiratory failure in critically ill patients [19,20]. To plan the phases of each educational session, Kolb’s Experiential Learning Theory was used as a reference theoretical framework [22]. In this study, a modified version of the abovementioned teaching model was applied and investigated. The differences between the two models are reported in Figure 1.

**Figure 2:**
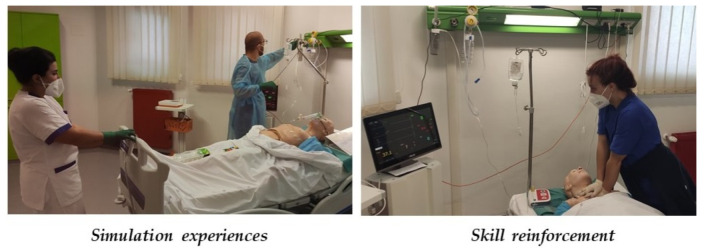
Students during simulation sessions and skill reinforcement.

After attending lectures on acute respiratory failure, students participated in a first 2 h guided teaching activity, which included a briefing (15 min), a high-fidelity simulation session (20 min), a first debriefing (15–20 min), and a skill reinforcement session (30 min); afterward, students were involved in a second high-fidelity simulation session related to the same scenario (20 min) and a final debriefing (15–20 min) (see Figure 2). The scenario was based on a left basal pneumonia clinical case derived from real data.

To guarantee learning retention over time, a ‘spaced learning’ approach was adopted with a more complex version of the first scenario proposed in a second learning module carried out 15 days later, in which arrhythmia was added to left basal pneumonia [23]. In each simulation session, two or three students were randomly involved. Inspired by the scaffolding educational process [24], the faculty provided personalized support. Accordingly, in the first simulation session, students practiced what they were able to do and, under the guidance of an expert faculty, identified areas for improvement through the guided video-assisted debriefing. The three-step debriefing process was conducted in a dedicated room. In the first step, faculty recapped the learning objectives and presented the aim of the debriefing. After that, a reflection period was provided to allow students to explore their deep reactions to the simulation experience. Finally, during the video-analysis phase, students had the opportunity to refine their understanding and attitudes, with a focus on current best practices. The debriefing was conducted in a blended manner, combining guided reflection and providing appropriate feedback.

Afterward, during the skill reinforcement phase, faculty provided assistance, allowing students to improve the abilities in which they were shown to be weaker (e.g., bag-mask ventilation or supraglottic airway management) and learn new skills that they would actively practice in the subsequent simulation session. This phase was also planned to facilitate students’ abstract conceptualization with analysis of concepts and active experimentation of key skills and actions; in this phase, students also had the opportunity to plan how to act during the next concrete experience (the second session) considering their gained competencies.

### 2.3. Variables, Data Collection Tools, and Procedures

At baseline, a semi-structured questionnaire was used to collect the following sociodemographic characteristics: sex (male/female), age (years), upper-secondary school attended (classical/science or other schools), upper-secondary school grade (total), employment as a nurse (yes/no), employment in a critical care ward (yes/no), Basic Life Support and Defibrillation Certification (yes/no), and previous participation in a high-fidelity simulation learning experience (yes/no).

The four Kirkpatrick levels for evaluating training programs (results, behaviors, learning, and perceptions) were used as the reference framework to assess the impact of the modified educational model experimented in this study [25]. Its ultimate intent consisted of enabling participants to improve respiratory failure outcomes in critically ill patients (results). To achieve this goal, students needed to possibly change their current behaviors and apply advanced nursing skills in clinical practice (behaviors). However, the prerequisite was that they must know what to do and how to do it, which means that they must achieve knowledge, skills, and positive attitudes (learning) to promote new clinical behaviors. Furthermore, to be motivated to learn, students must favorably react to the educational model and demonstrate a high level of satisfaction and positive lived experiences (perceptions).

According to the abovementioned Kirkpatrick framework, an adaptation of the Italian 10-item General Self-Efficacy Scale [26], the 13-item Student Satisfaction and Self-Confidence in Learning [27], and an ad hoc Group Performance Structured Checklist based on the guidelines of the Italian Society of Anesthesiology, Analgesia, Resuscitation, and Intensive Therapy [28] were used to document the learning outcomes.

Agreeing to Bandura’s theoretical framework, self-efficacy is a person’s belief in their ability to successfully execute the behavior required to produce the expected outcome [29]. The level of self-efficacy referred to a specific clinical scenario that can impact future behaviors of participants when they are faced with similar clinical conditions. The adapted General Self-Efficacy Scale is a four-level Likert instrument (1 = not at all true; 4 = totally true) that provides a score ranging from a minimum of 10 to a maximum of 40 points [19,30]. A higher self-efficacy score denotes greater student’s belief to be able to cope with a similar clinical problem (respiratory failure).

Satisfaction in learning is the perception of students about the teaching experience. Investigating satisfaction is important since a positive perception of simulation activities can motivate students to learn [25].

Self-confidence refers to an individual’s perception about their level of sureness to be equal to the task [31]. Like self-efficacy, the level of self-confidence was considered a proxy of behavior application in clinical practice.

The Student Satisfaction and Self-Confidence in Learning is a five-level Likert instrument (1 = strongly disagree; 5 = strongly agree) designed to measure students’ satisfaction with the simulation activity (five items), as well as self-confidence about the skills practiced and knowledge about caring for the type of patient presented in the simulation (eight items). This scale provides a score ranging from five to 25 for satisfaction and eight to 40 for self-confidence. For both scales, a higher score denotes greater satisfaction and self-confidence in learning. 

The 20-item performance checklist identified all key actions that nurses were expected to perform when caring for a patient in a life-threatening clinical condition, such as acute pneumonia or arrhythmia. For each action performed by a group during the simulation, one point was assigned, leading to a maximum achievable performance score of 20.

In this study, any possible change in the learning and perceptions of students was exclusively observed.

Following a phenomenological approach, an audio-recorded face-to-face in-depth interview was utilized to document the lived experience of participants. The interview was guided by a series of questions utilized in a previews research experience [20].

Data related to self-confidence and self-efficacy were collected before (T1 and T3) and after (T2 and T4) each educational experience. The performance was measured independently by two faculty members in the control room during each simulation session. Any disagreement was resolved by rewatching the video recordings. The satisfaction in learning was measured at the end of each leaning experience (T2 and T4).

### 2.4. Ethics

This study was conducted in accordance with the Declaration of Helsinki [32]. Approval was obtained only from the board of the graduate intensive care nursing course of the University of L’Aquila, because the training interventions were part of the normal educational program. Before using data for research finality, the study aims were explained to students, and their written informed consent was obtained. No students refused to provide their consent. However, students were free to participate and were assured of no consequences on their academic program in case of refusal. Confidentiality of data was guaranteed in accordance with the Italian law.

### 2.5. Data Analysis

Data were summarized using frequencies (*n*), percentages (%), central tendency indices (mean and median), and dispersion measures, such as standard deviation (SD), interquartile range (IQR), and range (min–max). The normal distribution of continuous data was tested using the Shapiro–Wilk test and visually assessed using histograms, boxplots, and Q–Q plots. Differences in the magnitude of students’ learning outcomes were explored using the nonparametric Wilcoxon signed-rank test, since a non-normal distribution was revealed. The possible association between some of the participants’ characteristics (employment in a critical care ward, Basic Life Support and Defibrillation Certification, and previous participation in a high-fidelity simulation learning experience) and learning outcomes was explored using the *U* Mann–Whitney test. The statistical significance was fixed at *p* < 0.05. All data were analyzed using IBM SPSS version 25.0 (IBM Corp., Armonk, New York, NY, USA).

With regard to qualitative data analysis, two of the authors independently transcribed the recorded interviews. Afterward, the experience of participants was documented through the following essential steps: (1) bracketing, (2) intuiting, (3) analyzing, and (4) describing [33]. In the first step, researchers abstained from expressing their opinions and making evaluations and judgments about students’ experiences. In the second step, they read the interview transcripts several times to gain a deep sense of the lived experiences. In the third step, researchers identified the essence of students’ experiences, and the most significant statements were extracted. Finally, the essential relationships in the statements were captured to pull out themes representing the essence of the lived experiences. In the abovementioned framework, qualitative data were synthesized according to the Giorgi phenomenological descriptive approach [34]. No software was used to handle the data.

## 3. Results

### 3.1. Participants

A total of 21/24 students agreed to participate in the study, with 20 completing both learning sessions (Table 1).

Most of them were female (17; 85.0%), and the mean age was 25.8 ± 2.6 years (median = 24.5; IQR = 4; min–max = 23–33). Overall, 60.0% of nurses (*n* = 12) had obtained a classical or science diploma before their bachelor’s degree in nursing. On average, the upper-secondary school grade was 75.9/101 ± 9.0 (median = 75.0; IQR = 14; min–max = 60–92), while the final bachelor’s in nursing grade was 105.1/111 ± 7.1 (median = 107.5; IQR = 11; min–max = 88–111). While attending their educational program, all the students also worked as nurses. Most of them (15; 75.0%) had no previous educational experience utilizing high-fidelity simulations before those proposed in the intensive care nursing course.

### 3.2. The Impact of the Modified Teaching Model on Students’ Learning Outcomes

**Figure 3:**
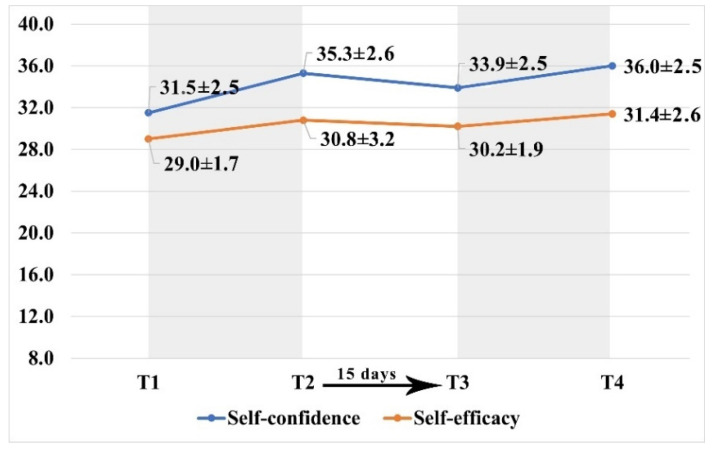
Self-confidence and self-efficacy over time.

Timepoint scores of self-confidence and self-efficacy are shown in Figure 3.

Immediately after the two teaching modules, a significant increase in the average level of self-confidence (T2 Δ = +3.8 ± 3.0, *p* ≤ 0.001; T4 Δ = +2.0 ± 2.5, *p* ≤ 0.003) and self-efficacy (T2 Δ = +1.8 ± 2.8, *p* = 0.010; T4 Δ = +1.2 ± 2.0, *p* = 0.016) was observed. When comparing the baseline levels of these variables with values obtained after both educational sessions (T1 and T4), an overall average gain was detected in self-confidence (Δ = +4.4 ± 3.1, *p* = 0.001) and self-efficacy (Δ = +2.4 ± 2.9, *p* = 0.004). After the 15 days that separated the first session from the second, a significant slight drop in the self-confidence level was detected (Δ = −1.4 ± 2.7, *p* = 0.038).

**Figure 4:**
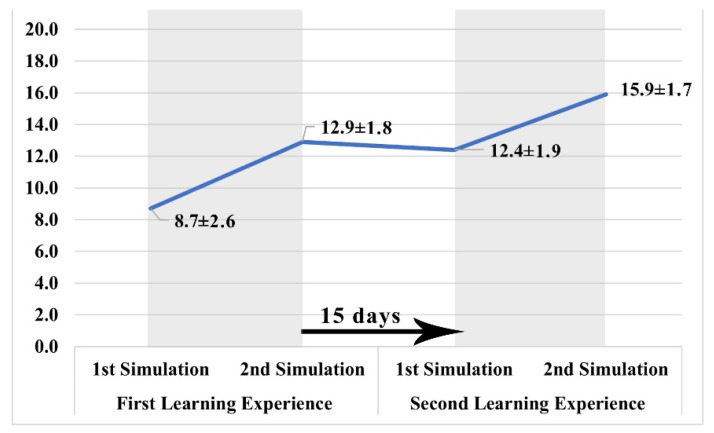
Performance during simulation sessions.

The modified learning model led to an increase in nurses’ performances (Figure 4). A significant performance average gain was detected during both the first (Δ = +4.5/20 ± 1.6, *p* ≤ 0.001) and the second training sessions (Δ = +3.5/20 ± 1.1, *p* ≤ 0.001).

An overall average gain was found when comparing the nurses’ performances demonstrated during the last simulation session with those showed in the first one (Δ = +7.2/20 ± 3.6, *p* ≤ 0.001). No significant loss in nurses’ performance was observed after the 15 days that separated the first and second training sessions (Δ = −0.5/20 ± 2.7, *p* = 0.422).

While nurses who recently (≤2 years) obtained the Basic Life Support and Defibrillation Certification showed a significant higher overall gain in self-efficacy than those who obtained the certification more than 2 years ago (Δ = +2.9, *p* = 0.017), no significant association was found between learning gains and the other students’ characteristics.

### 3.3. Satisfaction in Learning and Lived Experiences of Participants

Participants’ levels of satisfaction after the first and second simulation experiences (T2 and T4) were similar (24.0/25.0 ± 1.2 and 24.4/25.0 ± 1.2, respectively; *p* = 0.163).

Seventeen students agreed to participate in the face-to-face interviews, 14 of whom were female (82.4%). The thematic analysis revealed a total of four main themes and seven categories (Table 2).

### 3.4. Pleasant Discovery

High-fidelity simulations and their novel application modality were not clearly known by nurses until they were involved in it. Nevertheless, even if most of them had low expectations about the realism of the scenario and environment, as well as the power of simulation to promote learning, they made a pleasant discovery when they took part in the simulation.

‘[…] *having never worked with high-fidelity mannequins, I didn’t expect that they would be so realistic. I could monitor any parameter, talk to the patient, see him breathe, verify the correctness of the maneuvers; let’s say that this was the most interesting part, which I least expected*’

(interview #13).

### 3.5. Useful Learning Experience

The educational experience based on repeated simulations was perceived by participants as useful for their learning. The usefulness of this method was linked to the opportunity the nurses had to fill their knowledge gaps.

‘[…] *extremely useful, I feel like I have learnt new nursing skills* […]. *Particularly in the second experience, I filled some remaining learning gaps’*

(interview #2).

The experience also improved the nurses’ technical and nontechnical skills.

‘[…] *very useful, I’m satisfied because simulations enabled me to improve nursing skills that I have never had the opportunity to apply in clinical practice’*

(interview #10).

In addition, it gave them the opportunity to engage in simulated caring for critically ill patients. Importantly, they had the opportunity to reflect about their own clinical practice.

‘[…] *I lived an incredible experience! The simulation showed me how I should work in certain circumstances and allowed me to reflect about what I need to change in my clinical practice’*

(interview #7).

### 3.6. Emotional Change

Repeated exposures to high-fidelity simulation produced emotional changes in the nurses. Before the simulation experience, nurses reported an overload of negative feelings. Anxiety, fear, and embarrassment were the emotions mainly experienced. During the simulation sessions, nurses experienced emotional changes, albeit with different timing. The overload of negative emotions initially persisted, subsequently giving way for positive thoughts and emotions. Nurses felt gratified at the end of the learning sessions.

‘[…] *Surely anxiety, especially the first time! I did not know what to expect, I had never participated in simulations with so realistic scenarios, but when I got involved, I enjoyed it, I found it stimulating, and I would do it many more times’*

(interview #9).

‘[…] *First of all, anxiety and fear of not being able to handle the whole situation. And then, during simulations, I began to realize what I knew how to do. At the end, a lot of satisfaction, especially in the second* *round’*

(interview #1).

‘[…] *I felt embarrassed to be observed!* […] *during both the first and the second experience, the simulation made me understand many aspects of clinical practice, and this was very gratifying’*

(interview #8).

### 3.7. Active Learning Process

The nurses were of the opinion that repeated exposure to high-fidelity simulation improved their professional skills. In this regard, the simulation allowed nurses to apply their theoretical knowledge into practice in a safe way.

‘[…] *to repeat the same scenario twice and analyze our performance with the faculty helped a lot. With the faculty advice, our manual skills improved, and this helped us to consolidate our knowledge from a practical point of view*’

(interview #11).

The simulation also activated a self-analysis process that helped nurses to improve their clinical competence.

‘[…] *Surely, in this experience we reviewed what we did correctly and what had to be perfected. We understood how to work, basing our clinical practice on guidelines, protocols, and evidence*’

(interview #13).

‘[…] *it has certainly contributed understanding my own limits and the mistakes that I fail to consider in the workplace but are essential to consider* […]’

(interview #7).

## 4. Discussion

The aim of this study was to examine the impact of a modified teaching model based on multiple exposures to high-fidelity simulations on both the learning outcomes and the perceptions of graduate nurses enrolled in an intensive care nursing course. The educational model was appreciated by students as they expressed a high level of satisfaction and positive lived experiences. They simultaneously considered the model as a pleasant discovery and a useful experience. Even if these perceptions are not new in the nursing literature [1,19,20,35,36], at the early stage of the study, students expressed a low level of expectation toward the simulation activities. Considering that the use of high-fidelity simulations is not homogeneously embedded in nursing educational programs in Italy [37] and most of the participants had no previous experience with this technology, it is easy to understand why they had misconceptions and low expectations of the method. However, the perception of a high-level of realism during simulation sessions and learning opportunities offered by the high-fidelity simulator positively surprised students, creating the best conditions for learning [25]. Ultimately, a positive influence of multiple exposures to high-fidelity simulations on learning outcomes was observed. Interestingly, while students progressed in their learning experience, their learning outcomes significantly improved. The learning spiral embedded in our educational model, inspired by Kolb’s experiential learning framework, can help us to understand this result [22]. In this regard, the initial concrete experience allowed students to develop early an overall understanding of both skills and attitudes needed to effectively face the clinical emergencies. In this phase, students’ performances were not expected to be excellent, and a margin for improvement was expected. The subsequent debriefing and skill reinforcement stages were aimed at providing nurses with new nursing skills to effectively manage the more complex clinical scenario they experienced in the second concrete experience. As nurses had the opportunity to repeat the scenario, they showed a rapid and significant improvement in their skills. In this regard, it is known that multiple repetitions help students to focus themselves on educational objectives and facilitate learning, especially when the educational path presents, as in our case, new and more complex clinical scenarios [6,38]. The short time interval between learning sessions (15 days) probably also contributed to retention of the learning gain and considerable improvements in the achievement of the overall educational outcomes. Considering the persistent lack of knowledge about the ideal time interval to use when educational sessions are spaced over time, this study could represent a contribution to the development of knowledge about this topic [19,23,39].

According to the nurses, this experience allowed them to fill their knowledge gaps and acquire advanced skills through a self-analysis of their clinical activities and applying theory into practice. They described the experience as an active learning process since it improved their awareness of how to act in certain circumstances, gave them the chance to revise their fieldwork in line with clinical guidelines, and facilitated the beneficial evolution of learning outcomes [40].

As reported in a previous research experience, multiple exposures to high-fidelity simulations led students to experience an emotional change from negative to positive emotions [20]. At the early stage of the study, students had negative feelings, such as anxiety, fear, and embarrassment, which are recognized as potentially detrimental to learning [41]. However, multiple exposures to simulations and tailored support given during sessions [42] allowed participants to quickly absorb their initial emotions and focus on the learning outcomes by appreciating the support given by the faculty. In addition, repeated simulations allowed students to make mistakes without consequences and progressively improve their orientation during the simulation activities. These elements favored the perception of a psychologically safe learning environment [43] that facilitated the emotional change and provided the basis for an optimal learning experience for participants. Furthermore, nurses reported approaching an unexpected, highly realistic, immersive experience that enhanced their engagement level and fostered the learning gain. The level of realism and its perception by nurses are recognized as fundamental to achieving behavioral, emotional, and cognitive engagement, in order to translate knowledge, skills, and attitudes into clinical practice [44].

A significant higher overall gain in self-efficacy was detected in participants who recently (≤2 years) obtained the Basic Life Support Certificate. Even if the small sample size does not allow definitive conclusions to be drawn, this result highlighted the need for further studies aimed at exploring the role of potential sources of influence of graduate nursing students’ learning outcomes.

The multimethod design adopted in this study helped to expand and foster understanding of the deep meaning of results, thus providing a valuable contribution to the current debate about the best application modality of simulations in graduate critical care nursing courses. Despite the positive contributions of this study, the limited sample size, the explorative nature, and the absence of a control group call for caution in generalizing the results to other educational settings. In addition, a follow-up study to evaluate the behaviors adopted by the participants in clinical practice is not foreseen. It is important that future studies evaluate, according to the third and fourth levels of the Kirkpatrick training evaluation framework, whether the learning gains obtained through simulation can be transformed into clinical behaviors and impact the health outcomes of critically ill patients.

## 5. Conclusions

A theoretical-based, 2 h teaching model characterized by a double simulation session (briefing, simulation, and debriefing), separated by a supervised skill reinforcement stage, with ideally two participants, and repeated 15 days later can be considered beneficial to the learning outcomes of graduate nurses enrolled in an intensive care program.

## Figures and Tables

**Table 1:** Participants’ characteristics (*n* = 20).

	Mean (SD)	*N*	%
**Sex**			
Female		17	85.0
Male		3	15.0
**Age**			
Years	25.8 (2.6)		
**Upper-secondary school**			
Classical studies or science education		12	60.0
Other upper-secondary school		8	40.0
**Final upper-secondary school grade**			
Points (60–101)	75.9 (9.0)		
**Final degree program grade**			
Points (66–111)	105.1 (7.1)		
**Working as a nurse**			
Yes		20	100.0
No		0	0.0
**Working in a critical care ward**			
Yes		14	70.0
No		6	30.0
**Basic Life Support and Defibrillation Certification**			
Yes		18	90.0
No		2	10.0
≤2 years		9	45.0
>2 years		9	45.0
**Previous high-fidelity simulation experience**			
Yes		5	25.0
No		15	75.0

**Table 2:** Thematic analysis summary.

Themes	Categories
1. Pleasant discovery	1. Low expectations
2. Useful experience	2. Effective learning
	3. Critical reflection
3. Emotional change	4. Negative feelings overcome
	5. Feels gratified
4. Active learning process	6. Application of theory into practice
	7. Self-analysis

## Data Availability

Data are available on reasonable request to the corresponding author.
